# The interaction of breastfeeding and genetic factors on childhood obesity

**DOI:** 10.1016/j.eurox.2024.100334

**Published:** 2024-08-09

**Authors:** Mahsa Danaie, Maryam Yeganegi, Seyed Alireza Dastgheib, Reza Bahrami, Fatemeh Jayervand, Amirhossein Rahmani, Maryam Aghasipour, Mohammad Golshan-Tafti, Sepideh Azizi, Zahra Marzbanrad, Ali Masoudi, Amirmasoud Shiri, Mohamad Hosein Lookzadeh, Mahmood Noorishadkam, Hossein Neamatzadeh

**Affiliations:** a Department of Obstetrics and Gynecology, Iran University of Medical Sciences, Tehran, Iran; b Department of Obstetrics and Gynecology, Iranshahr University of Medical Sciences, Iranshahr, Iran; c Department of Medical Genetics, School of Medicine, Shiraz University of Medical Sciences, Shiraz, Iran; d Neonatal Research Center, Shiraz University of Medical Sciences, Shiraz, Iran; e Department of Plastic Surgery, Iranshahr University of Medical Sciences, Iranshahr, Iran; f Department of Cancer Biology, College of Medicine, University of Cincinnati, Cincinnati, OH, USA; g Department of Pediatrics, Islamic Azad University of Yazd, Yazd, Iran; h Shahid Akbarabadi Clinical Research Development Unit, School of Medicine, Iran University of Medical Sciences, Tehran, Iran; i Department of Obstetrics and Gynecology, Firoozgar Hospital, Firoozgar Clinical Research Development Center, Iran University of Medical Sciences, Tehran, Iran; j General Practitioner, Shahid Sadoughi University of Medical Sciences, Yazd, Iran; k General Practitioner, Shiraz University of Medical Sciences, Shiraz, Iran; l Mother and Newborn Health Research Center, Shahid Sadoughi University of Medical Sciences, Yazd, Iran

**Keywords:** Breastfeeding, Genetic factors, Epigenetics, Childhood, BMI, Obesity

## Abstract

Childhood obesity represents a pressing global public health concern due to its widespread prevalence and its close connection to early-life exposure to risk factors. The onset of obesity is contingent upon the interplay of genetic composition, lifestyle choices, and environmental as well as nutritional elements encountered during both fetal development and early childhood. This paper critically examines research discoveries in this area and concisely outlines the influence of breastfeeding on genetic predispositions associated with childhood obesity. Studies have demonstrated that breastfeeding has the potential to reduce childhood obesity by impacting anthropometric indicators. Moreover, the duration of breastfeeding is directly correlated with the degree to which it alters the risk of childhood obesity. Current explorations into the link between genetic factors transmitted through breast milk and childhood obesity predominantly focus on genes like FTO, Leptin, RXRα, PPAR-γ, and others. Numerous research endeavors have suggested that an extended period of exclusive breastfeeding is tied to a diminished likelihood of childhood obesity, particularly if sustained during the initial six months. The duration of breastfeeding also correlates with gene methylation, which could serve as the epigenetic mechanism underpinning breastfeeding's preventative influence against obesity. In summary, the thorough evaluation presented in this review underscores the intricate nature of the association between breastfeeding, genetic factors, and childhood obesity, providing valuable insights for future research efforts and policy formulation.

## Introduction

The rapid advancement of the global economy and significant lifestyle changes have led to a notable increase in childhood overweight and obesity worldwide [1], [2]. Previously, childhood obesity was not as common and was not seen as a major public health concern. However, it has now become a significant global health challenge that endangers children's well-being [3]. In 1980, the rates of overweight and obesity in boys and girls in developed nations were 16.9 % and 16.2 % respectively. These figures rose to 23.8 % and 22.6 % by 2013 [4]. Numerous studies have shown that childhood obesity, especially in adolescents, is a major risk factor for adult obesity [5]. A retrospective analysis involving more than 1000 individuals found that nearly half of those who were obese as adults had developed obesity during childhood, leading to metabolic syndrome conditions such as hypertension, high triglyceride levels, abnormal insulin metabolism, and hyperinsulinemia [6]. Conversely, individuals who did not experience childhood obesity had a lower likelihood of developing these conditions [7]. As children's BMI reaches the 85th to 95th percentile range, the risk of adult obesity increases [8], [9]. Research has also indicated that individuals who were overweight during adolescence face an increased risk of cardiovascular diseases and other chronic non-communicable diseases in adulthood, even if they lose the excess weight later in life [10]. Therefore, preventing obesity in children and adolescents is crucial in reducing the incidence of adult obesity.

Numerous lines of evidence support the recommendation of breastfeeding until children reach the age of two to reduce the risk of overweight and obesity [11]. The early months after birth are crucial due to rapid development and a doubling of body weight within 4–6 months [12]. Research shows that breastfeeding duration is significantly linked to lower BMI levels, with each additional month corresponding to an average decrease in BMI of about 0.04 and 0.03 kg/m2 among children aged 1–2 and 3–4 years, respectively [11], [13]. Moreover, each month of extended breastfeeding is associated with a decrease in waist circumference of 0.06 cm among children aged 12–24 months [14]. A meta-analysis of high-quality studies revealed that breastfeeding is linked to a 13 % decrease in the likelihood of overweight or obesity [15]. Therefore, feeding type plays a vital role in promoting healthy growth and preventing overweight and obesity during this stage of life [16]. Additionally, breastfeeding protects against obesity and adiposity in adolescence and adulthood by improving anthropometric indices and biochemical parameters [17]. The preventive impact of breastfeeding on childhood and adolescent obesity can indirectly help reduce the occurrence of obesity and related disorders in adulthood.

Obesity is a chronic metabolic disorder impacted by diverse elements, such as genetic and environmental, although in rare cases, genetic factors alone may be responsible [18]. The heritability of Obesity ranges from 40 % to 80 %, indicating a strong genetic influence on the risk for obesity [19], [20], but the current obesogenic environment can exacerbate the genetic risk for obesity [3], [21], [22]. However, given that genetic factors are unlikely to change significantly over a short period, it's important to consider the impact of behavioral and environmental factors on the rapid rise in obesity rates in recent years [21], [23]. These factors include changes in the environment that promote obesity, alterations in behavior and lifestyle, changes in food supply and consumption patterns, and reduced physical activity [24], [25]. While genetic differences are key determinants of individual weight variation and susceptibility to obesity, these susceptibilities can be influenced by environmental factors [17], [24]. Several lines of evidences have suggested that breastfeeding can modify the impact of obesity-related genes by regulating DNA methylation levels and the expression of high-risk alleles, offering protection against obesity and related non-communicable diseases [26], [27], [28], [29]. Studies have determined that the duration of breastfeeding is linked to variances in the DNA methylation patterns of genes such as NRF1, FTO, and LEPR, which play a role in the regulation of gene expression and metabolism [30], [31]. Breastfeeding additionally exerts its influence early in life by postponing the occurrence of the peak and rebound of adiposity, thereby assisting in the prevention of overweight and obesity among children with a heightened genetic susceptibility [11], [32]. In this study, we have conducted a comprehensive and in-depth examination of the interplay between breastfeeding and genetic factors with regards to their impact on the occurrence of childhood obesity, with particular emphasis on the impact of gene polymorphisms.

## FTO gene

The FTO is a key gene identified through genome-wide association studies (GWAS) that contributes to polygenic obesity in people of diverse ethnic backgrounds, including both children and adults [33]. The FTO gene plays a crucial role in cell proliferation and differentiation through the PI3K/Akt signaling pathway, and it may also be involved in the interaction between 5′ AMP-activated protein kinase (AMPK) and the PI3K/AKT/mTOR pathway [1], [34], [35], [36]. Multiple studies have highlighted the role of the FTO gene, particularly the rs9939609 polymorphism within the first intron, in increasing BMI and adiposity [37], [38]. This gene may have the potential to disrupt the hypothalamic regulation of appetite, energy expenditure, and metabolic rate, with various factors capable of influencing its effects [39]. In the RAINE cohort study, Abarin and colleagues observed that an extended period of exclusive breastfeeding was linked to a reduced body mass index (BMI) in individuals carrying the risk allele of the FTO rs9939609 gene. This effect was achieved through the regulation of the FTO gene and the adjustment of energy equilibrium during infancy. The research demonstrated a lower in BMI by 0.12 and 0.18 kg/m2 for each additional month of breastfeeding in the AT and AA groups, respectively, suggesting that those with the AA genotype are more susceptible than those with the AT genotype [40]. da Silva and colleagues proposed a notable correlation between FTO rs9939609 variations and anthropometric measurements as well as dietary intake. It was observed that children possessing the A/A genotype demonstrated an elevated body mass index in contrast to their T/A genotype counterparts [41]. The Avon Longitudinal Study of Parents and Children (ALSPAC) findings indicate that exclusive breastfeeding influences the trajectory of BMI and the age at which peak BMI occurs in children with different FTO genotypes [31], [42]. In 2017, Wu et al., revealed that exclusive breastfeeding for a duration of ≥ 5 months significantly reduced the risk of obesity in children carrying the FTO rs9939609 risk A allele. Furthermore, the study demonstrated that breastfeeding modified the age at which peak fat and fat accumulation occurred. In comparison to the non-breastfeeding group, children breastfed for 5 months experienced a delay of 2 to 3 months in reaching peak fat, while girls reached fat accumulation at a delayed by 6 months. At age 15, exclusively breastfed children had a predicted BMI decrease of 0.56 kg/m2 for boys and 1.14 kg/m2 for girls [43]. Another polygenic investigation revealed that prolonged exclusive breastfeeding for more than 5 months has the potential to decrease BMI of children with high obesity genetic risk scores at the age of 18 [11]. On average, boys experienced a lower of 1.14 kg/m2, while girls experienced a lower of 1.53 kg/m2. The health benefits of exclusive breastfeeding have the potential to counterbalance between 39 % and 70 % of the genetic risk of obesity [31], [44]. According to the findings of Cheshmeh et al., the expression level of the FTO gene was significantly higher in formula-fed and mixed-fed infants compared to breastfed infants. The expression level of the FTO gene in breastfed infants was significantly lower compared to the formula-fed and mixed-fed infants groups [45], [46].

The impact of feeding type on the expression level of obesity-related genes has been reported, and it appears that breastfeeding may possess a potential protective factor against development of obesity [47], [48]. Kanders et al. found a significant moderating effect of breastfeeding on the association between the rs9939609 variant and BMI. Their findings revealed that the FTO rs9939609 variant is adaptable, responding to both positive and negative environmental conditions in relation to BMI among adolescents. Individuals with the rs9939609 AA genotype were found to be more susceptible than those with the AT and TT genotypes to both shorter breastfeeding durations, which were associated with higher BMI, and longer breastfeeding durations, which were associated with lower BMI. Breastfeeding duration is likely one of several factors that influence the relationship between the rs9939609 variant and BMI. The FTO rs9939609 AA genotype may possess a plasticity variant with varying susceptibility to environmental influences [27]. It was observed in a Greek cohort [49] that breastfeeding moderated the association between the rs17817449 and rs9939609 polymorphisms in the FTO gene and adiposity, although these findings were not replicated in the ALSPAC cohort [31]. Horta et al., conducted a longitudinal study of a birth cohort in a city located in southern Brazil, spanning a duration of 30 years. Their findings showed that the duration of breastfeeding significantly influenced the relationship between a particular genetic variant in the FTO gene and the levels of body fat observed in adulthood. Their findings revealed that among infants who were breastfed for less than one month, all outcomes showed a steady increase in values with each additional copy of the A allele in the FTO rs9939609 polymorphism. In the case of individuals breastfed for one month or more, the associations tended to align in the same direction, but with reduced magnitude and less consistency across all outcomes. Notably, interactions demonstrated significant p values (≤ 0.05) for BMI, fat mass index, and waist circumference. This suggests that even among young adults, breastfeeding plays a moderating role in the relationship between the FTO variant rs9939609 and body composition [50]. The observation that breastfeeding moderates the adipogenic effect of the rs9939609 variant allele has also been reported in other contexts, providing further support to the idea that breastfeeding may impact the development of adiposity later in life, given the association between the FTO gene and food intake [51]. This hypothesis has also been put forth in other analyses of gene-nvironment interactions involving breastfeeding, genetic variants in the FADS2 gene, and intelligence [52].

## Leptin gene

Leptin is a protein hormone primarily produced by adipose tissue, and it plays a crucial role in regulating energy balance, appetite, and metabolism. It acts on the hypothalamus in the brain, where it binds to specific receptors and initiates a complex signaling cascade that ultimately influences food intake and energy expenditure. The production and secretion of leptin are directly proportional to the amount of adipose tissue in the body, with higher levels of leptin corresponding to increased fat mass. This relationship allows leptin to serve as an important feedback signal, informing the brain about the body's energy stores and triggering appropriate physiological responses to maintain energy homeostasis. The LEP gene was first discovered and explained in 1994, and the human LEP gene is situated on chromosome 7q31.3 and comprises three exons and two introns, spanning approximately 20 kb in length [53]. Leptin is produced and secreted in breast milk, especially in colostrum, and is expressed in the mammary gland of lactating women [54]. Numerous research studies have highlighted the influence of the mother's BMI on the levels of leptin in breast milk during pregnancy and childhood obesity [55], [56]. Logan et al. conducted a study that uncovered a non-linear relationship between maternal BMI and the concentration of leptin in breast milk at the 6th week, 6th month, and after one year of breastfeeding [57]. Conversely, Miralles et al. studied 28 non-obese women and found no significant correlations between the concentration of leptin in breast milk and the body weight of newborns [58]. Similarly, Khodabakhshi et al. conducted a study that revealed no statistical correlation between the concentration of leptin in breast milk and the occurrence of overweight or obesity in children [59]. Savino et al. found higher levels of leptin in the serum of breast-fed newborns compared to formula-fed newborns, particularly during the first month of life [60]. Leptin, which exerts its effects in early life, has the potential to influence the risk of obesity later in life. Some studies have explored the link between breastfeeding and the methylation levels of genes associated with obesity in offspring [61]. Prolonged breastfeeding promotes increased expression of the LEP gene and an elevation in plasma leptin concentration [62]. The duration of breastfeeding has been linked to changes in DNAm that affect cell signaling systems, development of anatomical structures and cells, and the development and function of the immune and central nervous systems [63]. Results from a cross-sectional study involving children in the Netherlands revealed a negative association between the duration of breastfeeding and the methylation level of the LEP gene in 17-month-old children [64]. Sherwood et al. found that a longer duration of breastfeeding is associated with a decreased methylation level of the LEP gene when children reach the age of 10. Their findings showed an 8.17 unit decrease in the expression level of the Leptin cg23381058 CpG site for each additional week of breastfeeding, influencing the association between the LEP gene and children's BMI at the age of 10 [62], [65]. The most recent research findings validate the Obermann-Borst et al. discoveries in the study conducted in the Netherlands during a later phase of childhood (10 years) [62], although not extending into young adulthood (18 years) [66]. Reduced methylation levels of the LEP gene result in enhanced gene expression, thus suppressing hunger. This could potentially explain how breastfeeding helps prevent childhood obesity [67].

## PPAR-γ gene

The human peroxisome proliferator-activated receptor gamma (PPAR-γ) plays a crucial role in regulating lipid and glucose levels, adipocyte differentiation, lipid storage, and insulin sensitization [68]. Due to its importance in macronutrient metabolism, extensive research has been conducted on this gene to explore its potential involvement in obesity, particularly in identifying significant gene-environment interactions [69], [70]. Within the coding region of the PPAR-γgene, there is one common polymorphism (Pro12Ala) as well as 16 rare missense and nonsense mutations that have been extensively examined for their connection to obesity and associated metabolic disorders [71]. Verier et al., found a significant interactive effect between breastfeeding and PPAR-γgene polymorphisms on childhood obesity. Their findings revealed that among children carrying the high-risk phenotype Pro12Ala of the PPAR-γgene, BMI, waist circumference, and skinfold thickness all decreased with an increased duration of breastfeeding compared to children who were artificially fed. However, for children with the non-high-risk phenotype Pro12Pro of the PPAR-γ gene, breastfeeding and its duration did not significantly influence obesity-related indicators between the breastfeeding and artificial feeding groups [72]. The absence of a clear dose-response effect seen in the investigation of the PPAR-γ Ala12 allele remains uncertain; one possible explanation is that the influence of breastfeeding on the programming process is mainly influenced by interactions between genes and nutrients during infancy, rather than being a quantitative phenomenon linked to the duration of exposure. However, their results were consistent with those of Mook-Kanamori et al., showing that the Ala12 allele was associated with increased weight gain in early infancy in non-breastfed children [73]. A multitude of mechanisms has the potential to elucidate how breast-feeding may counteract the detrimental influence of the Ala12 allele in childhood. The impact of PPAR-γ genotypes on the relationship between dietary fat and BMI has been demonstrated, taking into account the fact that breast milk represents a diet with a distinct fat consumption [72], [74]. Furthermore, there exists a potential hypothesis which posits that breast milk or the act of breast-feeding provides elements such as prostaglandin J_2_, a naturally occurring PPARγ ligand. Consequently, the observed lower in PPARγ2 transcriptional activity among individuals carrying the Ala12 allele might be mitigated by the consumption of breast milk [72], [75]. Thus, this results suggests that the potential effectiveness of breastfeeding in preventing and controlling childhood obesity may only manifest in children with specific genetic backgrounds.

## IGF-I gene

Increased protein consumption, particularly of branched chain amino acids (BCAA: leucine, isoleucine, and valine), triggers the release of insulin and insulin-like growth factor I (IGF-I) [76]. This process amplifies the function and specialization of adipocytes in the first two years of life through an autocrine-paracrine pathway [77], [78]. Savino et al. demonstrated a direct link between IGF-I and weight, as well as body mass index [60]. Conversely, individuals fed with formula exhibited higher levels of IGF-I and urinary C-peptide [39]. In contrast, artificial feeding resulted in elevated IGF-I concentrations in the perinatal period and reduced serum levels in adults [79], [80]. Current investigations on the impact of small for gestational age and breastfeeding on childhood obesity primarily concentrate on high molecular weight adiponectin and IGF-I. Adiponectin primarily aids in glucose and lipid metabolism, while high molecular weight adiponectin mainly promotes glucose and lipid metabolism. IGF-I plays a crucial role in regulating growth and development during childhood. Elevated IGF-I levels can lead to increased body weight and BMI [81], [82]. A birth cohort study in Spain found that breastfed small for gestational age infants at four months of age had lower levels of high molecular weight adiponectin content and IGF-I content compared to formula-fed counterparts. These levels were similar to those of breastfed infants appropriate for gestational age. Studies have identified a lower in IGF-I and glucagon-like peptide-1 levels by 9 mg/L and 9 pmol/L, respectively, as well as a decrease in the muscle-to-fat ratio by 0.89 after small for gestational age infants were breastfed at four months of age. However, Ong and colleagues did not support these findings. Martin et al. recently provided evidence that breastfeeding has no impact on IGF-I concentrations [83].

## RXRA gene

RXRA regulates lipid metabolism by inhibiting the accumulation of triglycerides, cholesterol, and non-esterified fatty acids through the CD36 signaling network [84]. The research by Pauwels and colleagues showed that a one-month increase in breastfeeding duration led to a 0.217 % rise in RXRA CpG2 methylation levels. Additionally, higher levels of RXRA methylation were linked to infant weight loss at one year of age. Other studies found that exclusive breastfeeding affected the relationship between 13 CpG sites in girls, 2 CpG sites in boys, and children's BMI [61]. Children breastfed for 3 to 5 months showed changes in overall DNA methylation levels at age 6, slowing the growth rate of BMI compared to non-breastfed children. Pauwels et al. also noted that children breastfed for 4–6 months had significantly lower RXRA methylation compared to non-breastfed children, as well as those breastfed for 1–3 months, 7–9 months, or 10–12 months. Their findings concluded that each additional month of breastfeeding led to a 0.217 % increase in RXRA CpG2 methylation [67]. Methylation of the RXRA gene in humans is negatively linked to the duration of breastfeeding [67]. After 12 months of breastfeeding, there were notable differences in infant growth and methylation of the buccal RXRA and LEP genes. A positive correlation was observed between RXRA CpG2 methylation and breastfeeding duration. Methylation levels for CpG3 and CpG islands of the RXRA gene were significantly lower in children breastfed for 4–6 months compared to those who were not breastfed (only CpG3), as well as those breastfed for 7–9, 10–12, or 1–3 months. Additionally, children breastfed for 7–9 months (6.1 %) exhibited higher LEP CpG3 methylation compared to those breastfed for 1–3 months (4.3 %) and 10–12 months (4.6 %). Moreover, children breastfed for 10–12 months had a much lower weight [85]. In 2022, Chavira-Suárez et al., found that maternal obesity leads to dysregulation of circulating steroid hormones in obese mothers and low fetal weight. This modifies DNA methylation of the RXRα gene, as well as RXRα mRNA and protein expression in the umbilical cord in a sex-dependent manner [86].

## ACAC-B gene

The ACAC-B gene is responsible for encoding acetyl-CoA carboxylase beta, a vital component in the process of biosynthesis and oxidation of fatty acids. This gene plays a crucial role as the step that restricts the rate of mitochondrial fatty acid oxidation, accomplishing this by carboxylating acetyl-CoA into malonyl-CoA [87]. Obese individuals exhibit a greater abundance of triglycerides within their muscles in comparison to non-obese individuals. Additionally, an adaptive mechanism occurs in response to heightened adipose tissue, whereby the expression level of ACAC-B within adipose tissue decreases [88], [89]. A study showed that breastfeeding, compared to formula feeding and mixed feeding, leads to a decrease in the expression level of the ACAC-B gene [17]. Ma et al. reported that the ACAC-B rs2268388 polymorphism was associated with BMI in the general population and obesity in patients with type 2 diabetes. This SNP can also affect gene expression in adipose and hepatic tissue. The authors proposed that ACAC-B plays a crucial role in obesity and may contribute to lipid metabolism abnormalities in individuals with T2DM-associated nephropathy [88], [89]. The ACAC-B gene has been shown to significantly contribute to the development of obesity and diabetes by reducing fat oxidation. However, breastfeeding during infancy can help mitigate the negative consequences of the ACAC-B gene in adulthood.

## Epigenetic impacts of breastfeeding

The epigenetic mechanism may act as a protective factor against obesity [90]. The biological components in human milk have epigenetic effects by triggering mechanisms such as histone modification, DNA methylation, and chromatin remodeling [91]. These active compounds can decrease or increase the expression of many critical genes in metabolic pathways, which could help prevent weight gain and subsequent non-communicable diseases later in life [92]. Epigenetic modifications, including DNA methylation, non-coding RNA regulation, and histone modifications, are currently key areas of research revealing the interaction between genes and the environment in childhood obesity pathogenesis [90], [92]. Among these, DNA methylation research is the predominant focus in this field. Substantial evidence indicates that adverse environmental and behavioral exposures can significantly impact children's obesity and metabolism-related risk genes' methylation levels, leading to alterations in gene expression levels [93], [94]. These changes can have synergistic or antagonistic effects with genetic risks [95]. For instance, a mother's diet during pregnancy can alter the DNA methylation level of the CpG island of the WNT5B gene (cg23757341) in fetal umbilical cord blood, influencing gene expression levels, subsequently affecting children's adipogenesis and insulin secretion [96]. Several research has also indicated that breastfeeding can impact key genes in the obesity pathway, reducing the expression level of FTO and carnitine palmitoyltransferase IA (CPT1A) genes while increasing the expression level of the PPAR-α gene in 5–6-month-old infants [13]. The GAS5 gene is involved in metabolic processes and the regulation of the immune system. As a significant epigenetic regulator, GAS5 also has potential as a component of breast milk, which could have a significant impact on breastfeeding outcomes [63]. The International Epigenetics in Pregnancy and Childhood Working Group conducted an international collaborative study on children's gene methylation due to environmental exposure during pregnancy, integrating methylation research data from multiple birth cohort projects [97]. This effort aims to unveil the gene-environment interaction in childhood obesity, significantly advancing the understanding of the mechanism. Additionally, histone modifications represent potential mechanisms for gene-environment interaction. A single histone can undergo modifications involving multiple chemical groups, such as methylation and acetylation, thereby activating or repressing gene expression [92], [98]. While no studies have reported the role of histone modifications in epigenetic changes in obese children, animal experiments provide insights for further research [99]. For example, after mice are fed a high-fat diet, levels of H3K9 me2 or H3K9 me3 in the mouse gene promoter decrease, and a series of genes activated by histone modifications, such as MAP3K5, MET, and VEGFA, are up-regulated. KEGG Enrichment analysis results are associated with pathways related to lipogenesis, energy metabolism, and inflammation [100].

## MicroRNA

MicroRNA (miRNA), a non-coding RNA molecule, can regulate gene expression by inhibiting messenger RNA (mRNA) translation or causing mRNA degradation [101]. The distinct microRNA profile of human breast milk, including exosomal microRNAs, plays a role in regulating genes responsible for various immunological and physiological functions [85], [102]. Research indicates that miRNA can be influenced by external environmental factors and respond to them. For example, breastfeeding can stimulate CpG demethylation of intron 1 of the FTO gene through the miRNA-29b/miRNA-21 signaling pathway, resulting in increased expression of the FTO gene [45], [103]. A study on obese male adolescents found that after 6 weeks of exercise combined with dietary intervention, the level of miRNA-126 significantly increased [104]. Animal experiments have also demonstrated that intrauterine risk factors such as excessive weight gain during pregnancy can impact the metabolic signaling pathways of offspring through the regulation of miRNA-126 [105]. Programmed overexpression of miRNA-126 leads to insulin resistance in children, thereby increasing the risk of obesity and T2DM in offspring [106], [107].

## Conclusion

Breastfeeding can modify multiple genes linked to obesity, and when combined with other environmental factors, it may help prevent childhood weight gain. The recommendation to exclusively breastfeed for the first six months is the best approach to ensure healthy physical growth in infants and prevent childhood obesity. However, more research is needed to determine the impact of breastfeeding on key obesity-related genes and the molecular pathways involved in childhood obesity. Further exploration of the long-term effects of breastfeeding on obesity-related genes and pathways is crucial for developing targeted interventions and public health strategies to reduce childhood obesity. Understanding the complex interaction between breastfeeding, genetic predisposition, and environmental factors will provide valuable insights into preventing and managing obesity in children. Additionally, investigating the epigenetic mechanisms through which breastfeeding influences gene expression related to obesity will contribute to the development of personalized approaches for addressing childhood weight issues.

## Ethics approval

This article does not include any studies involving human participants or animals conducted by any of the authors.

## Funding

There is no funding source.

## Consent for publication

None.

## CRediT authorship contribution statement

**Seyed Alireza Dastgheib:** Conceptualization. **Zahra Marzbanrad:** Writing – review & editing. **Ali Masoudi:** Visualization. **Amirmasoud Shiri:** Writing – review & editing. **Maryam Yeganegi:** Conceptualization. **Mohamad Hosein Lookzadeh:** Supervision. **Mahsa Danaie:** Conceptualization. **Maryam Aghasipour:** Writing – review & editing. **Mohammad Golshan-Tafti:** Funding acquisition. **Sepideh Azizi:** Writing – original draft. **Mahmood Noorishadkam:** Funding acquisition. **Hossein Neamatzadeh:** Conceptualization, Data curation. **Reza Bahrami:** Supervision. **Amirhossein Rahmani:** Methodology. **Fatemeh Jayervand:** Resources.

## Declaration of Competing Interest

The authors declare that they have no known competing financial interests or personal relationships that could have appeared to influence the work reported in this paper.
